# Biomimetic electrospun nanofibrous scaffold for tissue engineering: preparation, optimization by design of experiments (DOE), *in-vitro* and *in-vivo* characterization

**DOI:** 10.3389/fbioe.2023.1288539

**Published:** 2023-10-31

**Authors:** Shabnam Anjum, Ting Li, Dilip Kumar Arya, Daoud Ali, Saud Alarifi, Wang Yulin, Zhang Hengtong, P. S. Rajinikanth, Qiang Ao

**Affiliations:** ^1^ Department of Tissue Engineering, School of Intelligent Medicine, China Medical University, Shenyang, Liaoning, China; ^2^ NMPA Key Laboratory for Quality Research and Control of Tissue Regenerative Biomaterial, National Engineering Research Centre for Biomaterials, Institute of Regulatory Science for Medical Device, Sichuan University, Chengdu, Sichuan, China; ^3^ Department of Laboratory Medicine, Shengjing Hospital of China Medical University, Shenyang, Liaoning, China; ^4^ Department of Pharmaceutical Sciences, Babasaheb Bhimrao Ambedkar University, Vidya Vihar, Lucknow, India; ^5^ Department of Zoology, College of Science, King Saud University, Riyadh, Saudi Arabia

**Keywords:** electrospinning, PVP, PVA, subcutaneous implant, tissue engineering

## Abstract

Electrospinning is a versatile method for fabrication of précised nanofibrous materials for various biomedical application including tissue engineering and drug delivery. This research is aimed to fabricate the PVP/PVA nanofiber scaffold by novel electrospinning technique and to investigate the impact of process parameters (flow rate, voltage and distance) and polymer concentration/solvent combinations influence on properties of electrospun nanofibers. The *in-vitro* and *in-vivo* degradation studies were performed to evaluate the potential of electrospun PVP/PVA as a tissue engineering scaffold. The solvents used for electrospinning of PVP/PVA nanofibers were ethanol and 90% acetic acid, optimized with central composite design via Design Expert software. NF-2 and NF-35 were selected as optimised nanofiber formulation in acetic acid and ethanol, and their characterization showed diameter of 150–400 nm, tensile strength of 18.3 and 13.1 MPa, respectively. XRD data revealed the amorphous nature, and exhibited hydrophilicity (contact angles: 67.89° and 58.31° for NF-2 and NF-35). Swelling and *in-vitro* degradability studies displayed extended water retention as well as delayed degradation. FTIR analysis confirmed solvent-independent interactions. Additionally, hemolysis and *in-vitro* cytotoxicity studies revealed the non-toxic nature of fabricated scaffolds on RBCs and L929 fibroblast cells. Subcutaneous rat implantation assessed tissue response, month-long biodegradation, and biocompatibility through histological analysis of surrounding tissue. Due to its excellent biocompatibility, this porous PVP/PVA nanofiber has great potential for biomedical applications.

## Graphical Abstract

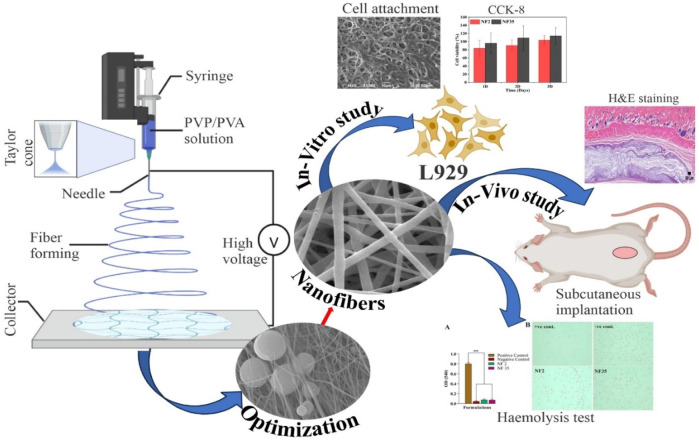

## 1 Introduction

In tissue engineering and regenerative medicine (TERM) research, selecting and optimizing a biomaterial that physiochemically replicates the extracellular matrix (ECM) is important (Mao et al., 2023). To mimic the ECM structure of the original tissues, materials must be biocompatible, biodegradable and possess the required physiochemical properties (Qiu et al., 2023; Xin et al., 2023). Tissue engineering offers crucial and viable tissue structures for the purpose of tissue replacement (Zhu et al., 2008; Pezeshki-Modaress et al., 2015; Quan et al., 2022).

Electrospun nanofibers play a key role in tissue regeneration owing to their multiple functionalities, such as high surface area, mechanical stability (stiffness and tensile strength), nanoscale architecture, interfibrous porous microstructure, sustained drug delivery and high scale-up potential. There is growing evidence that nanofibrous scaffolds are suitable for TERM because they can recapitulate ECM components, modulate cellular responses, and promote mineralization and osseointegration (Anand et al., 2022a; Yu et al., 2023). Electrospinning represents an innovative and effective method for creating biomimetic non-woven nanofibrous scaffolds (Anand et al., 2023). The electrostatic voltage is used as the driving force for the formation of nano-sized fibers from different materials, such as polymers, metals and ceramics. When exposed to a very high electrical potential, the charged polymer is captivated by the collector and forms fiber strands with porous structures.

The dimensions and morphological configuration of nanofibers produced through electrospinning are influenced by a range of factors. These factors can be categorized into three groups: attributes of the polymer solution (such as molecular weight, concentration, solvent, viscosity, conductivity, and surface tension), operational parameters (including applied voltage, flow rate, collector configuration, and tip-to-collector distance), and environmental conditions (encompassing temperature, atmospheric pressure, and humidity). These factors collectively impact the capability to spin and the morphology of electrospun nanofibers (Anjum et al., 2022). Hence, achieving the intended fiber structure necessitates the optimization and modeling of electrospinning variables. The influence of these variables on fiber shape has been extensively explored in various investigations carried out by numerous researchers.

Polyvinylpyrrolidone (PVP) stands as a significant amorphous polymer, characterized by its notable biocompatibility, high tensile strength, exceptional solubility in various organic solvents, minimal chemical toxicity, proficient spinnability, and non-hazardous nature. The process of electrospinning has found extensive application in transforming diverse materials into fibers, leveraging PVP’s spinnability and fiber extraction capabilities. Another synthetic polymer that exhibits a commendable aptitude for forming membranes, along with low toxicity in physiological environments and excellent biocompatibility, is polyvinyl alcohol (PVA) (Agarwal et al., 2021; Thakur et al., 2023). However, a notable drawback of semi-crystalline PVA is its susceptibility to suspension under physiological conditions. By amalgamating PVA and PVP, the constraints posed by this drawback can be surmounted, owing to the presence of inter-chain hydrogen bonds that bolster their stability (Mohammed et al., 2021; Pandey et al., 2023).

Response Surface Methodology (RSM) emerges as a significant approach in modeling the electrospinning procedure due to its capacity to account for the interplay among diverse parameters. RSM is an amalgamation of mathematical and statistical techniques employed in the experimental modeling and analysis of diverse input data that can impact specific outcomes or the quality aspects of a process. Notably, Gu et al., in their research, explored the quantitative correlation between electrospinning process variables and the distribution of average fiber sizes for gelatin and PVA nanofibers, utilizing RSM (Carvalho et al., 2009). In a separate study, Amiri et al. utilized RSM to fabricate chitosan-collagen nanofibers with minimized diameters through the utilization of electrospinning technology (Amiri et al., 2018). The significance of electric field strength was further explored by Jacobs et al. Through the utilization of a Box-Behnken design within the framework of RSM, they examined the influence of the solvent ratio (trifluoroacetic acid/dichloromethane) as well as interaction effects on the diameter of chitosan nanofibers (Jacobs et al., 2011). The aforementioned exploratory literature has demonstrated that a number of electrospinning parameters affect the fiber’s diameters and shape. However, further research is required to fully understand the connection between these variables and fiber structure. There has not been any publicized thorough study that closely examines the impact of numerous parameters. So far, there is limited or no data available in the literature that elucidates the connection between electrospinning parameters and fiber diameter for information and explanations. Additionally, there is an absence of preceding documentation concerning PVP/PVA electrospun nanofibers within varying solvents.

The primary objective of this study is to comprehensively investigate the impact of distinct solvents and process parameters on the diameter of PVP/PVA nanofibers utilizing the electrospinning technique. The research endeavors to optimize various system parameters including concentration, applied voltage (kV), nozzle-collector distance (cm), and flow rate (ml/h) concerning PVP/PVA nanofibers generated in both acetic acid and ethanol solvents. This optimization was conducted using DOE, version 13. RSM with central composite design (CCD) was used to model and optimize the electrospinning process to minimize the nanofiber diameter. The comparison of *in vitro* with *in vivo* degradation was also carried out. There are different sources of lipases in the human body such as leukocytes, present in the wound healing process, with the lipase concentration of healthy adults in the range of 30–190 U/L. Thus, the degradation of the PVP/PVA nanofibers were monitored both in phosphate buffered saline (PBS) and *in-vivo* subcutaneous rat implantation. The *in-vitro* degradation kinetics were evaluated through the quantification of weight loss, swelling degree and thermal behavior whereas *in-vivo* degradation was assessed by using histopathology. Biocompatibility study was investigated using L929 cell lines and hemolysis study.

## 2 Materials and methods

PVA (Mw: 80,000) and PVP (Mw: 90,000) were purchased from Macklin Biochemical Co. Ltd. (P.R. China). Cell Counting Kit-8 purchased from KeyGEN BioTECH (China). L929 cell lines were gift sample from professor Huang Zhongbing (Biomedical engineering collage; Sichuan university). All additional chemicals and reagents employed in the study were of analytical grade.

### 2.1 Design and optimization of PVP/PVA nanofiber

#### 2.1.1 Optimization of polymer concentration

DOE software version 13 was used to optimize the polymer concentration as well as their nanofibers producibility. In this study, we applied the RSM technique using a CCD to evaluate the influence of polymers solution concentration on nanofiber producibility in ethanol. Face centered central composite design (FCCD) with four center points was used to investigate the relationship between independent variables and responses. Accordingly, the percentage of polymers (%w/v) was considered as the process parameter in the DOE. Two levels, low (−1) and high (+1), were defined for each polymer with different concentrations. As shown in Table 1, 12 runs were performed and the nanofiber producibility scale range from 1 to 5 was measured as the response.

**TABLE 1:** DOE table for the optimization of polymer (PVP and PVA) concentration.

Nanofiber batches	Run	Factor 1	Factor 2	Response 1 NF producibility
		A: PVP (%w/v)	B: PVA (%w/v)	
NF1	1	12.00	5.00	4
NF2	2	8.00	2.00	2
NF3	3	8.00	5.00	2
NF4	4	4.00	2.00	1
NF5	5	8.00	5.00	2
NF6	6	4.00	8.00	1
NF7	7	12.00	2.00	3
NF8	8	4.00	5.00	1
NF9	9	8.00	5.00	2
NF10	10	8.00	8.00	3
NF11	**11**	**12.00**	**8.00**	**5**
NF12	12	8.00	5.00	2

The bold values represent has optimized batches.

#### 2.1.2 Optimization of electrospinning process parameters in different solvents

To analyze the effect of process parameters in different electrospinning solvents on the producibility and diameter of nanofibers, the selected batches were used for further studies. In this study, a CCD design was used to prepare and optimize the electrospun nanofibers. The main parameters including voltage (kV), flow rate (ml/hr) and distance (cm) with two levels, low (−1) and high (+1), were evaluated for optimizing the nanofibers. As shown in Table 2 and Table 3, 18 runs were performed and the nanofiber diameter was measured as the response transferred to the software. The main aim of the DOE was to determine the optimal conditions for fabricating nanofibers.

1) PVP/PVA in acetic acid solvent.
2) PVP/PVA in ethanol solvent.

**TABLE 2:** CCD design for the optimization of electrospinning process parameters in acetic acid.

Nanofiber batches	Run	Factor 1	Factor 2	Factor 3	Response 1
		A: Flow rate (ml/hr)	B: Voltage (kV)	C: Distance (cm)	Diameter (nm)
NF1	1	1.50	26.00	12.00	232.11
**NF2**	**2**	**2.50**	**26.00**	**8.00**	**179.224**
NF3	3	2.00	18.00	10.00	235.502
NF4	4	2.00	26.00	10.00	272.6
NF5	5	2.00	22.00	10.00	192.093
NF6	6	2.00	22.00	8.00	121.91
NF7	7	2.50	18.00	12.00	96.379
NF8	8	2.00	22.00	12.00	136.3
NF9	9	2.00	22.00	10.00	192.093
NF10	10	1.50	22.00	10.00	258.611
NF11	11	1.50	18.00	12.00	237.92
NF12	12	2.50	26.00	12.00	207.983
NF13	13	2.50	22.00	10.00	177.731
NF14	14	1.50	26.00	8.00	189.144
NF15	15	1.50	18.00	8.00	262.178
NF16	16	2.50	18.00	8.00	129.306
NF17	17	2.00	22.00	10.00	192.093
NF18	18	2.00	22.00	10.00	192.093

The bold values represent has optimized batches.

**TABLE 3:** CCD design for the optimization of electrospinning process parameters in ethanol.

Nanofiber batches	Run	Factor 1	Factor 2	Factor 3	Response 1
		A: Flow rate (ml/hr)	B: Voltage (kV)	C: Distance (cm)	Diameter (nm)
NF19	1	2.00	22.00	12.00	182.845
NF20	2	1.50	18.00	12.00	309.61
NF21	3	2.00	22.00	10.00	183.681
NF22	4	1.50	22.00	10.00	336.599
NF23	5	2.00	22.00	10.00	183.681
NF24	6	2.00	22.00	10.00	183.681
NF25	7	2.50	26.00	12.00	388.681
NF26	8	2.50	18.00	8.00	272.57
NF27	9	2.50	22.00	10.00	406.577
NF28	10	2.00	22.00	8.00	84.346
NF29	11	2.00	22.00	10.00	183.736
NF30	12	2.00	18.00	10.00	198.105
NF31	13	1.50	26.00	8.00	121.897
NF32	14	1.50	18.00	8.00	328.217
NF33	15	2.50	18.00	12.00	275.956
NF34	16	2.00	26.00	10.00	192.736
**NF35**	**17**	**2.50**	**26.00**	**8.00**	**271.065**
NF36	18	1.50	26.00	12.00	155.389

The bold values represent has optimized batches.

#### 2.1.3 Preparation of nanofibers by electrospinning

Polymer solutions were prepared in different solvents by mixing 12% PVP and 8% PVA in 90% acetic acid and distilled water under continuous stirring for 8 h to obtain a uniform solution. A mixture consisting of 12% PVP and 8% PVA solutions was meticulously combined in a 50/50 ratio through rigorous stirring to achieve well-blended compounds suitable for electrospinning. The same procedure was replicated using ethanol. These prepared solutions were introduced into a 10 ml syringe fitted with a stainless-steel needle possessing an inner diameter of 0.4 mm. By connecting a positive electrode to the needle, a high-voltage power source was employed. For the collection of nanofibers, a collector enveloped in aluminum foil was employed, positioned at a distance of 8–12 cm from the spinning electrode. To ensure controlled extrusion, the syringe pump’s flow rate was fine-tuned to 1.5–2.5 ml/h, while the solutions were pumped using a high electric voltage ranging from 18–26 kV.

### 2.2 Physio-chemical characterization

#### 2.2.1 Scanning electron microscopy (SEM) analysis

The morphology of the electrospun nanofiber batches was analyzed using a SEM model S4800 from Hitachi, Japan. In this investigation, nanofiber samples were affixed to SEM specimen stubs utilizing double-sided carbon tape. Subsequently, a sputter-coating process with Au-Pd was conducted for a duration of 70 s. The analysis was carried out under varying magnifications at an accelerating voltage. The software employed for assessing the nanofiber scaffold diameter was ImageJ. Measurements of fiber diameters were taken at multiple locations for accuracy (Anand et al., 2022b).

#### 2.2.2 Mechanical testing

The mechanical characteristics of the nanofibrous mats were assessed using a Universal Testing Machine (UTM) (YG005A; Baien Instrument China) with a load cell of 5 cN capacity and the standard followed was American Society for Testing and Materials (ASTM, D882). The nanofibrous samples (*n* = 3) were cut into 1 mm width and 20 mm length, placed between the two clasps, and subjected to tensile displacement a crosshead speed of 8 mm/min. The stress-strain curve was utilized to calculate the Young’s modulus, tensile strength, and elongation at the point of fracture for the samples.

#### 2.2.3 Hydrophilicity characterization by contact angle

The angle formed between the solid surface and the interface of the liquid/vapor is known as the contact angle. To assess the surface wettability, whether it is hydrophilic or hydrophobic, of the nanofibers, a water contact angle instrument (model JC 2000C1; POWEREACH China) was employed. In this process, the sample was initially positioned on a level surface, following which a water droplet was carefully dispensed onto it using a moving needle. The spherical image of the droplet was captured by a digital camera and projected onto a monitor. Subsequently, the contact angle formed between the droplet and the surface of the nanofibers was measured. To ensure accuracy, at least three measurements were taken at different locations on the film and then averaged for comprehensive data analysis.

#### 2.2.4 Fourier transform infrared spectroscopy (FTIR)

FTIR (NEXUS 670; NICOLET USA) spectrometer was used to determine the constituting functional groups of different nanofibers. The structural changes occurring during electrospinning, blending, coating etc. was scanned by FTIR, using standard KBr crystal at room temperature. The spectra were obtained in transmission mode over a wavenumber ranged from 4,000 to 400 cm^−1^ with a resolution of 4 cm^−1^ (Gupta et al., 2018).

#### 2.2.5 X-ray diffraction (XRD)

The crystalline arrangement of the nanofiber scaffolds were assessed using an X-ray diffractometer (model DX-1000; PHILIPS USA). The samples were positioned on quartz zero background holders and subjected to examination with a commercially available XRD system. For detection, a solid-state Germanium detector cooled with liquid nitrogen was utilized, operating with Cu k-alpha radiation at a current of 45 kV and 40 mA. XRD patterns were gathered within a 2θ range spanning from 5 to 60°, with a scanning rate set at 2° per minute (Pandey et al., 2022; Deepak et al., 2023).

#### 2.2.6 Thermo-gravimetric analysis (TGA)

TGA (TGA/DSC 2/1600-ThermoStar; METTLER TOLEDO Switzerland) was used to employed thermal stability of different nanofibers. In a nitrogen atmosphere, all samples were subjected to heating at a rate of 10°C per minute across a temperature range spanning from 25°C to 800°C.

#### 2.2.7 *In-vitro* degradation

The degradation characteristics of the nanofibers were assessed by monitoring the weight loss at various time intervals. Scaffolds measuring 1 × 1 cm^2^ were precisely weighed (W_i_) and then placed into 5 ml plastic tubes containing 4 ml of PBS solution with a pH of 7.4. These tubes were subsequently positioned within a shaking incubator set at 100 rpm and 37°C. The incubation medium was renewed on a weekly basis. At each predetermined time interval, the samples were dried until a constant weight was achieved (W_f_) (Li et al., 2023). The weight loss percentage (%) was calculated using the formula:

$$\mathrm{Weight loss}\left(\%\right)=\frac{W_{i}-W_{f}}{W_{i}}x100$$

Where *W*_*i*_ and *W*_*f*_ represents the initial weight of the sample and the final weight after degradation, respectively.

#### 2.2.8 Water uptake capacity

The water retention capacity of the scaffolds was assessed by calculating the swelling ratio. Initially, the samples were sectioned into 1 × 1 cm^2^ pieces, and their dry weight (W_d_) was determined using an electronic weighing balance and recorded. Subsequently, the samples were immersed in a PBS (pH 7.4) at room temperature. At specified time intervals, the samples were retrieved from the solution and positioned on tissue paper to eliminate excess water adhering to the nanofiber surface (Singh et al., 2023). The weight of the moist nanofiber (W_w_) was then promptly measured. All measurements were executed in triplicate, and the water uptake capacity was computed using the subsequent equation:

$$\%\text{Water uptake capacity}=\frac{W_{w}-W_{d}}{W_{d}}x100$$

Where W_w_ represents the weight of the wet nanofiber scaffold and W_d_ corresponds to the dried weight. A triplicate analysis was conducted, and the average value was adopted as the percentage of water uptake.

### 2.3 *In-vitro* compatibility test

#### 2.3.1 *In-vitro* hemocompatibility test

The assessment of scaffolds’ hemocompatibility involved the use of freshly collected blood samples from healthy rats. Blood samples (5 ml) were gathered into tubes coated with heparin and then subjected to centrifugation at 3,000 rpm, leading to the separation of plasma from red blood cell (RBC) pellets settled at the tube’s bottom. With careful removal of the supernatant, the introduction of PBS into the tube ensued. A subsequent centrifugation step was applied to resuspended RBCs for cell isolation. The subsequently purified blood cells were diluted to achieve a 25 ml volume, thus creating an RBC suspension. Following this, 0.5 ml of the RBC suspension was aliquoted into four 1.5 ml tubes. Among these, two tubes were designated as negative and positive controls, wherein 1 ml of PBS and water was added, respectively. The remaining two tubes were subjected to treatment with nanofibers (NF2 and NF35) sized 1 × 1 cm^2^. All tubes, including controls and treated samples, were then incubated at 37°C for a duration of 3 h. Upon the conclusion of this incubation period, all tubes underwent centrifugation at 3,000 rpm for 10 min. After incubation and centrifugation, 200 µL of the supernatant from all the samples was transferred to a 96-well plate, followed by the measurement of haemoglobin absorption at a wavelength of 540 nm (Ghorai et al., 2022). The following formula was employed to determine the percentage hemolysis of the samples.

$$\text{Hemolysis }\left(\%\right)=\frac{{OD}_{Sample}-{OD}_{Negative\;control}}{{OD}_{Positive\;control}-{OD}_{Negative\;control}}x100$$

Where OD_Sample_, OD_Negative Control_, OD_Positive Control_ represents the optical density (OD) of the samples, negative control and positive control.

Microscopic images of the RBC condition were also captured.

#### 2.3.2 *In-vitro* cell line study on L929

##### 2.3.2.1 Cell viability study

L929 fibroblast cells will be seeded into 96-well culture plates at a predetermined cell density of 1×10^5^ cells/well. The cells will be cultured under standard conditions using appropriate DMEM media, supplemented with 10% foetal bovine serum and 1% penicillin-streptomycin, at a temperature of 37°C and under an atmospheric condition of 5% CO2. Prior to conducting the cell experiments, nanofibers with a standard size of 6 cm^2^/ml will be sterilized using UV light and 70% alcohol.

To generate extract solutions, sterile nanofibers will be placed in culture medium (DMEM containing 10% FBS and 1% penicillin-streptomycin). These mixtures will be incubated in a constant temperature shaker at 37°C for 24 h. At the conclusion of the extraction period, samples will be retrieved and the resulting extract solutions will be maintained at 37°C during the subsequent cytotoxicity testing. L929 cells will be exposed to the nanofiber extract solution, and cell proliferation on various nanofibers will be quantitatively assessed using Cell Counting Kit-8 (CCK-8; KeyGEN BioTECH; China) assay at specified time points. The OD value will be measured at 450 nm using a spectrophotometric microplate reader. The experiment will be conducted in triplicate to ensure statistical validity. The acquired data will be analysed to ascertain the impact of nanofibers on L929 fibroblast cell viability on days 1, 3, and 5. Cell viability was calculated using the following formula:

$$\mathrm{Cell viability}\left(\%\right)=\frac{{OD}_{Scaffold}}{{OD}_{Control}}\times 100\%$$

##### 2.3.2.2 Cytocompatibility and cell adhesion test on L929 cell lines

The L929 cells (murine fibroblast) were cultivated in DMEM culture media supplemented with 10% fetal bovine serum and 1% penicillin-streptomycin. The cells were then incubated at 37°C in an environment with 5% CO_2_. The nanofibers were trimmed to dimensions of 2 × 2 cm^2^, after which they underwent a sterilization process involving exposure to UV light for 30 min and treatment with 70% alcohol. Following this, nanofiber-coated 12-well plates were utilized to seed 1×10^4^ cells per well, and subsequent incubation took place. On the third and fifth days, the cell culture media was removed, and the cells were rinsed with PBS (7.4), fixed using a 4% w/v paraformaldehyde solution for 30 min, and subjected to a dehydration process using ethanol (ranging from 10% to 100%). To enable microscopic mineralization analysis, the samples were coated with a layer of platinum using sputter coating and studied using SEM.

### 2.4 *In-vivo* study

#### 2.4.1 Subcutaneous implantation

All surgical procedures involving animals received approval from the Animal Use and Care Committee of Sichuan University. The nanofibers underwent sterilization through exposure to both 70% ethanol and UV light. Wistar rats, each with a weight ranging from 200 to 250 g, were selected for the animal experiments. These rats were divided randomly into three groups: the first group underwent surgery alone, the second group received PVP/PVA in ethanol (NF35), and the third group received PVP/PVA in acetic acid (NF2); each group comprised six rats. Before the surgical procedures, all experimental animals were anesthetized using chloral hydrate. The dorsal region of the animals was shaved and then sterilized using a solution of 70% ethanol. A sterile surgical blade was employed to create an incision on the dorsum of each animal. Subsequently, a subcutaneous pouch was formed, and an implant measuring 1 × 1 cm^2^ was inserted into this pouch. Following the polymer implantation, the incision was sutured using a non-absorbable surgical black braided silk thread.

#### 2.4.2 *In-vivo* biocompatibility study

At the end of 1 and 3 weeks, the rats were humanely euthanized. The regions where the implants were positioned were carefully collected; this encompassed both the complete PVP/PVA nanofiber and the adjacent tissue. The collected samples were then immersed in a 4% (w/v) paraformaldehyde solution for fixation, allowing them to be preserved overnight. To prepare the samples for further analysis, they underwent a dehydration process by passing through a series of alcohol baths with varying concentrations, followed by treatment with xylene. Subsequently, the samples were embedded in paraffin and sliced into sections that were 5 µm thick. These sections were mounted onto slides, facilitating histological staining and subsequent imaging.

### 2.5 Statistical analysis

The data from all experiments were presented in the form of mean values accompanied by standard deviations (S.D.). Analysis of the data was performed using OriginPro 2019b software. One-way ANOVA was utilized to assess the data across all experiments, and this was followed by the application of the Tukey test for *post hoc* analysis. Statistical significance was indicated by * for a *p*-value less than 0.05, ** for a *p*-value less than 0.01, and *** *p*-value less than 0.001.

## 3 Results

### 3.1 Effect of polymer concentration on nanofiber producibility

**FIGURE 1:**
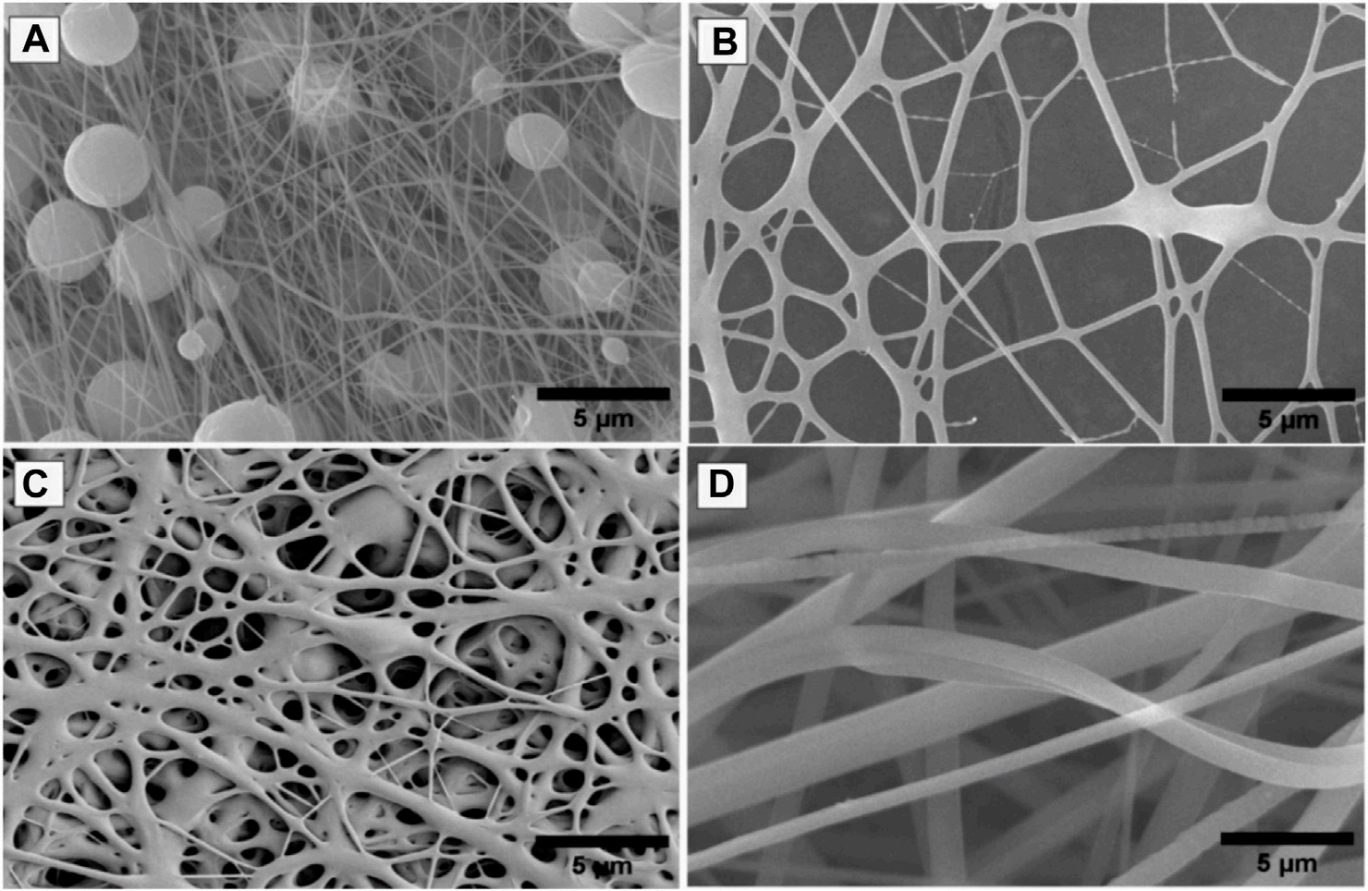
SEM images for nanofiber producibility to optimize the concentration of polymers, **(A)** Non-uniform nanofibers formed with beads, **(B)** Nanofiber formed but not smooth, **(C)** Non-uniform nanofibers formed with fewer beads, **(D)** Beads free uniform nanofibers formed.

During optimization, it was found that the polymer concentration plays a very important role in the formation of nanofibers; otherwise, nanofibers will form with numerous beads having no uniformity. The relationship between solution viscosity and polymer concentration is highly dependent on the concentration of polymer/solvent system. Each set of PVP/PVA was optimized by DOE for their concentration as mentioned earlier (Table 1), on the basis of their nanofiber producibility scale ranging from 1 to 5, which was measured as the response (Table 2; Table 3). SEM images for nanofiber producibility were represented in Figure 1.

**FIGURE 2:**
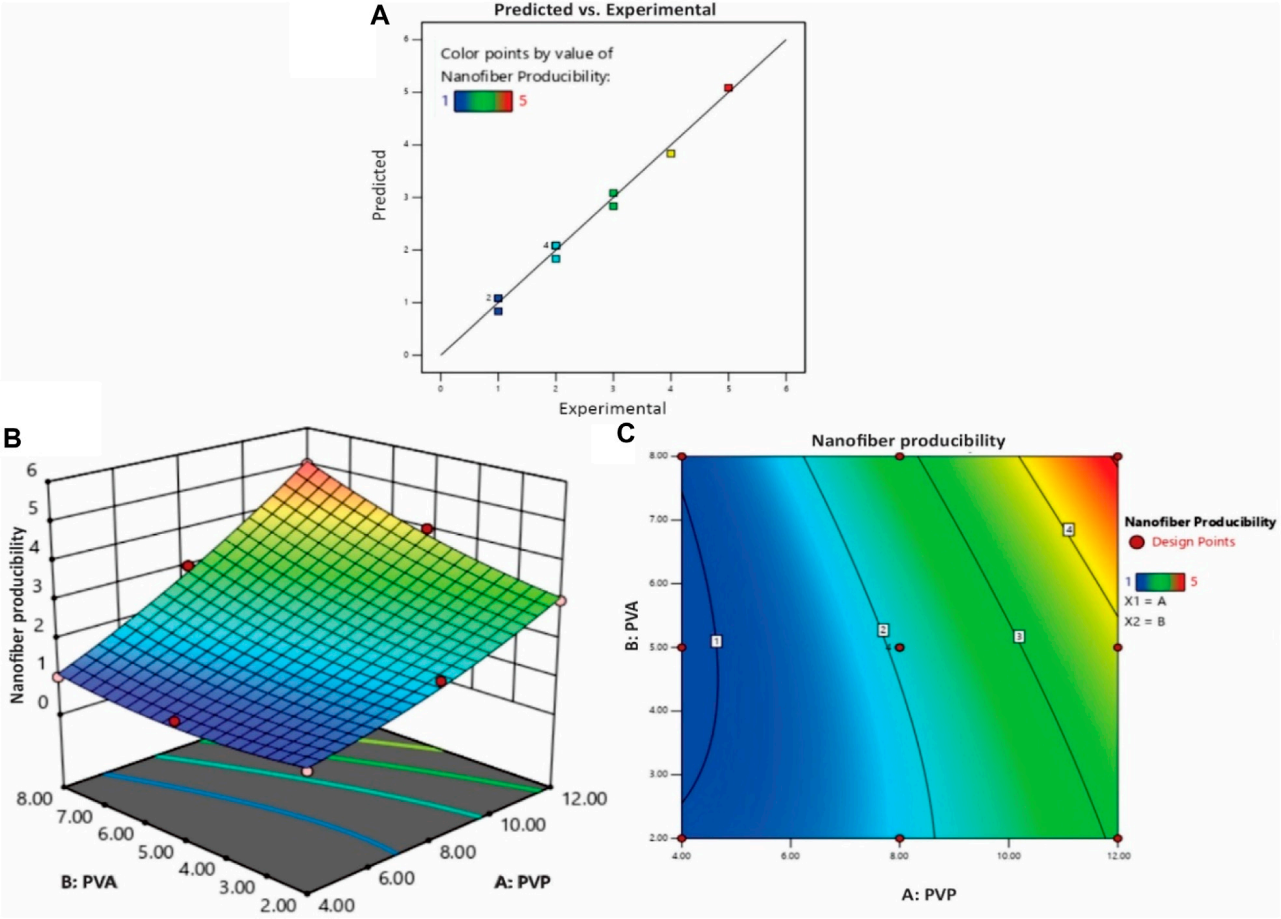
**(A)** The experimental versus predicted plot for optimizing polymer concentration (%) of PVP/PVA nanofibers and the effect of polymer concentration (%) PVP/PVA on the nanofiber production; **(B)** three-dimensional (3D) RSM plot and **(C)** Contour-plot.

As depicted in Figure 2A, the coordination between predicted and experimental values is commendable, encompassing a majority of the responses. This is further supported by the fact that all models possess AP (adequate precision) values surpassing 4, signifying a high level of precision. The NF11 batch was selected from (Table 1) which showed good nanofiber production whereas in other concentrations, polymers were failed to produce nanofibers due to high or low viscosity and lack of sufficient surface tension.

### 3.2 Effect of electrospinning process parameters

**FIGURE 3:**
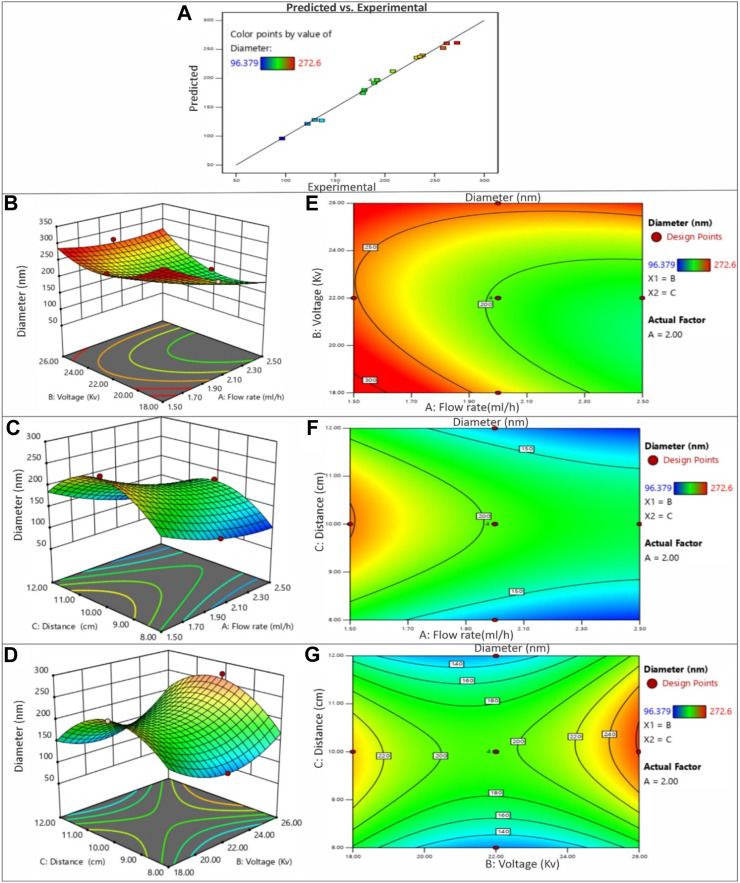
**(A)** The predicted versus experimental plot for average diameter of PVP/PVA nanofibers in acetic acid. Effect of process parameters on diameter of PVP/PVA nanofiber in acetic acid; **(B)** Effect of voltage and flow rate, **(C)** Effect of distance and flow rate, **(D)** Effect of voltage and distance -3D RSM and **(E)** Contour plot showing the effect of voltage and flow rate, **(F)** distance and flow rate and **(G)** voltage and distance on nanofiber diameter.

**FIGURE 4:**
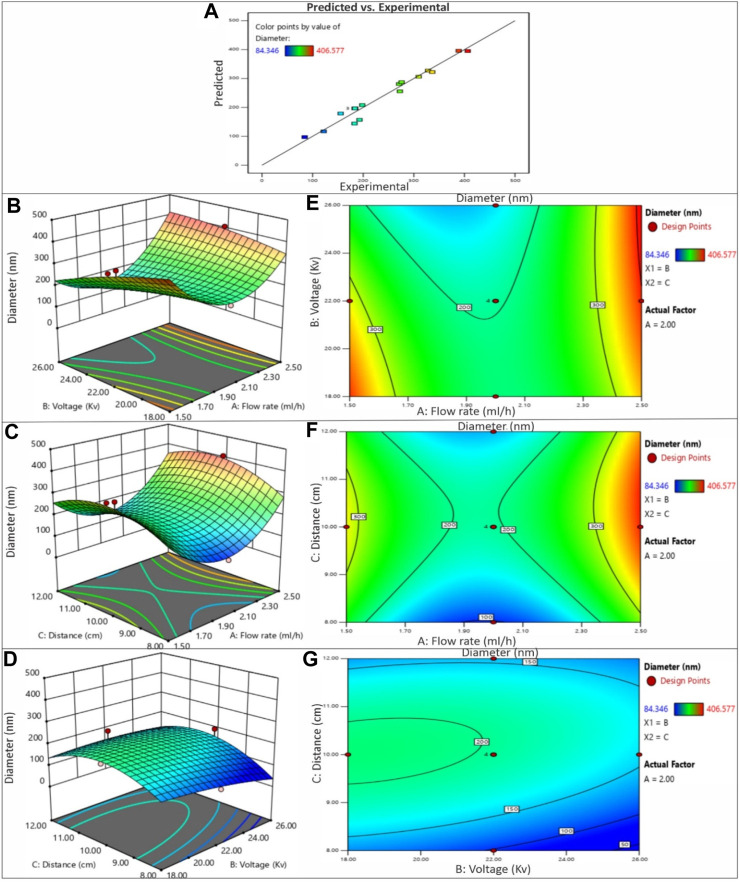
**(A)** The predicted versus experimental plot for average diameter of PVP/PVA nanofibers in ethanol solvent. Effect of process parameters on diameter of PVP/PVA nanofiber in ethanol; **(B)** Effect of voltage and flow rate, **(C)** Effect of distance and flow rate, **(D)** Effect of voltage and distance -3D RSM and **(E)** Contour plot showing the effect of voltage and flow rate, **(F)** distance and flow rate, **(G)** voltage and distance on nanofiber diameter.

**FIGURE 5:**
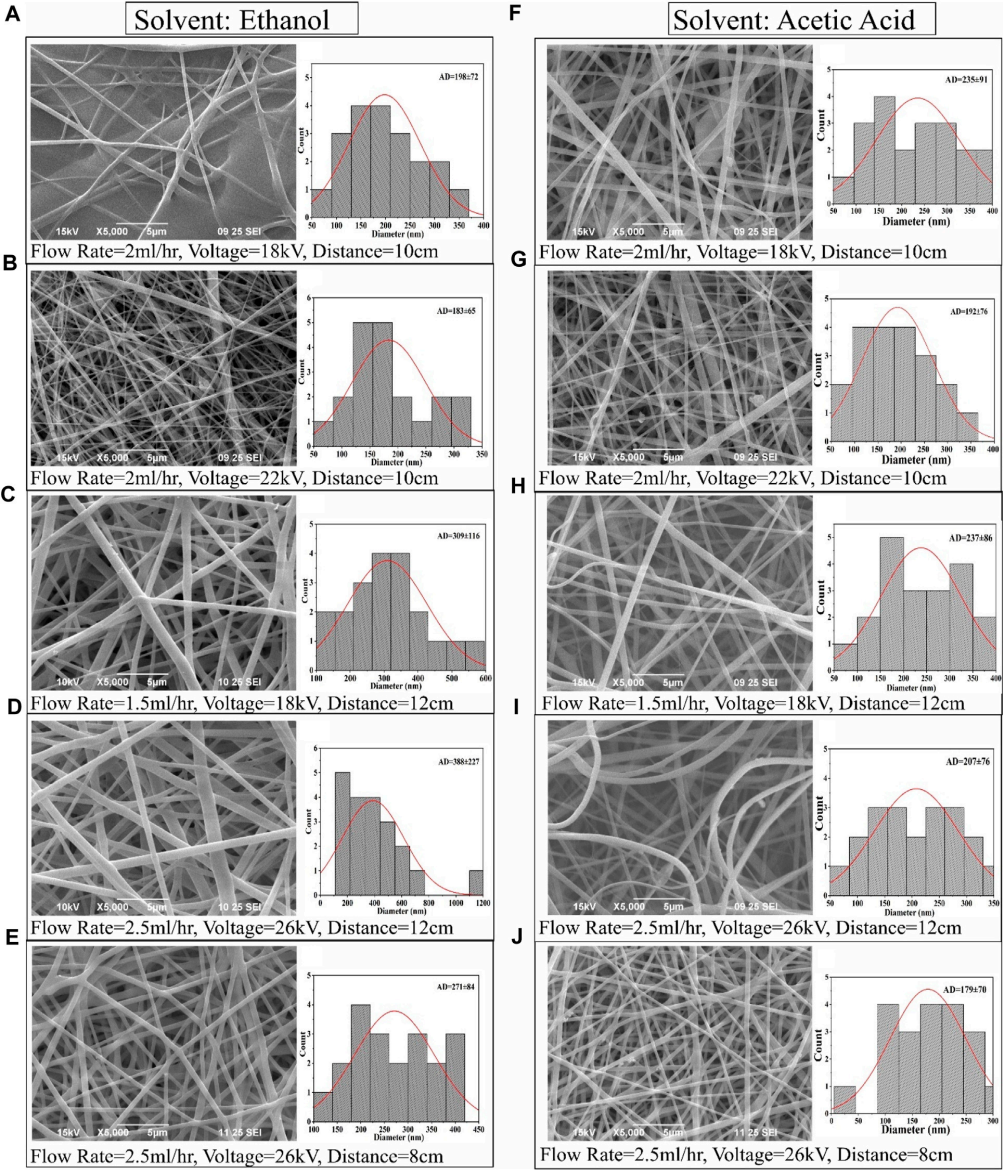
SEM images and corresponding fiber diameter distribution of PVP/PVA electrospun fibers in ethanol solvent **(A–E)** and acetic acid **(F–J)** at various electrospinning process parameter.

In this study, the primary objective was to achieve the production of uniform and smooth nanofibers by optimizing the electrospinning process parameters, namely distance, voltage, and flow rate, utilizing both acetic acid and ethanol solvents. To gain a deeper understanding of the process parameters and to establish a quantifiable relationship between the electrospinning process parameters and the resulting fiber diameter, the RSM was employed (Gu et al., 2005). By employing the DOE software, quadratic equations were generated to depict the governing relationships between the electrospinning parameters and the designated responses. The influences of diverse processing parameters such as applied voltage, flow rate, and distance, employed in distinct solvents, on all the generated nanofiber batches are presented in Figure 5. The contour and 3D plots, showcased in Figures 3, 4, aptly illustrate the high dependency of fibre diameter on the applied voltage, flow rate, and distance.

#### 3.2.1 Effect of voltage

The influence of voltage on fiber diameter remains a subject of debate and is markedly influenced by the specific characteristics of the polymer and solution employed. Elevated voltage levels can yield disparate outcomes in terms of fiber diameter alteration, as depicted in Figures 3, 4. The impact of heightened voltage on fiber diameter has prompted varying findings, with certain reports suggesting an augmentation in correlation with increased voltage for solutions involving PVA polymer (Zhang et al., 2005). Conversely, several studies focusing on the electrospinning of PVP have indicated a decrease in fiber diameter as voltage increases, attributed to the heightened repulsion force. Subsequently intensifying the electric field strength can lead to an expansion in diameter, likely due to the amplified electrostatic forces at play (Chuangchote et al., 2009). Elevated voltage levels facilitate a greater transport of fluid from the polymer solution. Moreover, they serve to expedite the exit of the polymer jet from the Taylor cone, thereby reducing the duration of the jet’s flight time (Miri et al., 2016). Enabling a greater polymer carriage and diminishing flight duration can contribute to the production of more substantial fibers at increased voltages. Nevertheless, heightened voltages concurrently amplify the electrostatic repulsion along the jet’s surface, potentially leading to a reduction in fiber diameter. Furthermore, exceedingly elevated voltage levels can induce the emergence of branched structures within the jets and fibers, ultimately yielding thinner fibers characterized by a broader diameter distribution (Garg and Bowlin, 2011). The impact of applied voltage and the distance between the nozzle and collector on the diameter of electrospun PVP/PVA nanofibers is twofold. In the case of an acetic acid solvent system, employing a shorter nozzle-collector distance and higher applied voltage leads to a reduced duration for both jet elongation and acetic acid solvent evaporation. Consequently, this combination encourages the production of smaller diameter PVP/PVA nanofibers. Conversely, within an ethanol solvent system, a higher applied voltage grants more time for the jet to elongate in the electric field and for the volatile ethanol solvent to evaporate. As a result, this configuration favours the formation of larger diameter nanofibers.

When examining the acetic acid solvent system at a constant nozzle-collector distance, augmenting the applied voltage leads to a decrease in the average diameter of PVP/PVA nanofibers. However, with a fixed applied voltage, elevating the nozzle-collector distance leads to a reduction in the average diameter (AD) of the nanofibers. This discrepancy can be elucidated by considering the relatively higher boiling point of acetic acid (118°C). In contrast, when using an ethanol solvent, decreasing the applied voltage or increasing the nozzle-collector distance diminishes the strength of the electric field, resulting in less acceleration and stretching of the jet. Consequently, this leads to the generation of larger PVP/PVA nanofibers during the electrospinning process. Subsequent to the dispersion and division of an unstable jet, solvents with lower boiling points, such as ethanol (78.37°C), swiftly undergo evaporation (Nasouri et al., 2015).

#### 3.2.2 Effect of flow rate

The rate at which the polymer solution flows within a given timeframe constitutes an additional element impacting the quality of nanofibers. As evidenced by the findings and outcomes presented in Table 2 and Table 3, employing lower flow rates yields nanofibers characterized by diminished and consistent diameters, attributed to the heightened charge density inherent in such conditions. It has been documented that elevating the flow velocity gives rise to the creation of nanofibers with larger diameters. The conjecture that nanofiber diameter decreases owing to amplified charge density at lower rates has also been put forth (Beachley et al., 2009). Furthermore, there are reports indicating that raising the flow rate leads to an augmentation in fiber diameter (Reneker and Chun, 1996). Furthermore, it is noteworthy that when the flow rate was decreased at 10 kV, the reduction in fibre diameters was more pronounced compared to the situation at 18 kV. This observation suggests that the impact of flow rate on fibre diameter is more conspicuous at lower voltages. Multiple studies have documented an upsurge in fibre diameter when flow rates are elevated. This correlation is rooted in the fact that increasing the flow rate enhances the solution volume available for electrospinning and the initial radius of the ejected jet. The resultant enlargement of the initial jet radius curtails bending instability and jet stretching, consequently leading to an increase in fibre diameter.

However, it is essential to note that an excessive flow rate not only augments nanofiber agglomeration but also gives rise to bead formation within the fibre structure due to insufficient time for solvent evaporation.

#### 3.2.3 Effect of distance

The distance between the nozzle and the collector constitutes an additional parameter influencing the regulation of nanofiber morphology and diameter. Achieving precise control over polymer solution evaporation before the fiber reaches the collector necessitates the optimization of this distance. Drawing insights from the outcomes and findings presented in Table 2 and Table 3, it becomes evident that the distance parameter is more closely tied to the applied voltage and flow rate. Extended distances have been associated with the generation of thinner nanofibers in accordance with reported results (Doshi and Reneker, 1995). Furthermore, the occurrence of beads becomes apparent when the distance between the nozzle and the collector is either excessively short or overly long (Yang et al., 2004). It has also been documented that reducing the distance to the collector can enhance the probability of fiber fusion. However, extending the distance beyond a certain threshold leads to a decrease in the strength of the electric field. At a specific point, this attenuation of field strength becomes notably significant (Ding et al., 2010).

### 3.3 Nanofiber morphology and diameter

Figure 5 illustrates SEM images of typical PVP/PVA electrospun nanofibers, offering insight into the diameter distribution of nanofiber samples. The average fibre diameter spanned from 100 to 400 nm. The SEM depictions of PVP/PVA nanofibers underscored the substantial influence of high voltage (18–26 kV), flow rate (1.5–2.5 ml/h), distance (8–12 cm), and solvent on fibre diameter. For instance, within an ethanol solvent using a flow rate of 2 ml/h, a voltage of 18 kV, and a distance of 10 cm, the resulting fibre exhibited an uneven surface. In contrast, under identical parameters but with an acetic acid solvent, a smooth and homogeneous nanofiber formation was observed.

A noteworthy observation entailed a minimal fibre diameter of 179.224 nm, achieved at a voltage of 26 kV, flow rate of 2.5 ml/h, and distance of 8 cm in the acetic acid solvent. Correspondingly, the average diameter of electrospun PVP/PVA nanofibers, measuring 271.065 nm, was attained using a voltage of 26 kV, flow rate of 2.5 ml/h, and distance of 8 cm in an ethanol solvent. Ethanol exhibited promise as a suitable solvent for PVP/PVA, yielding electrospun fibres with a broad range of diameter distribution conducive to microfiber production. Comprehensive analysis substantiated that polymer concentration, solvent selection, and process parameters all exerted noteworthy impacts on the resulting diameter values.

### 3.4 Physiochemical properties

#### 3.4.1 Tensile strength

**FIGURE 6:**
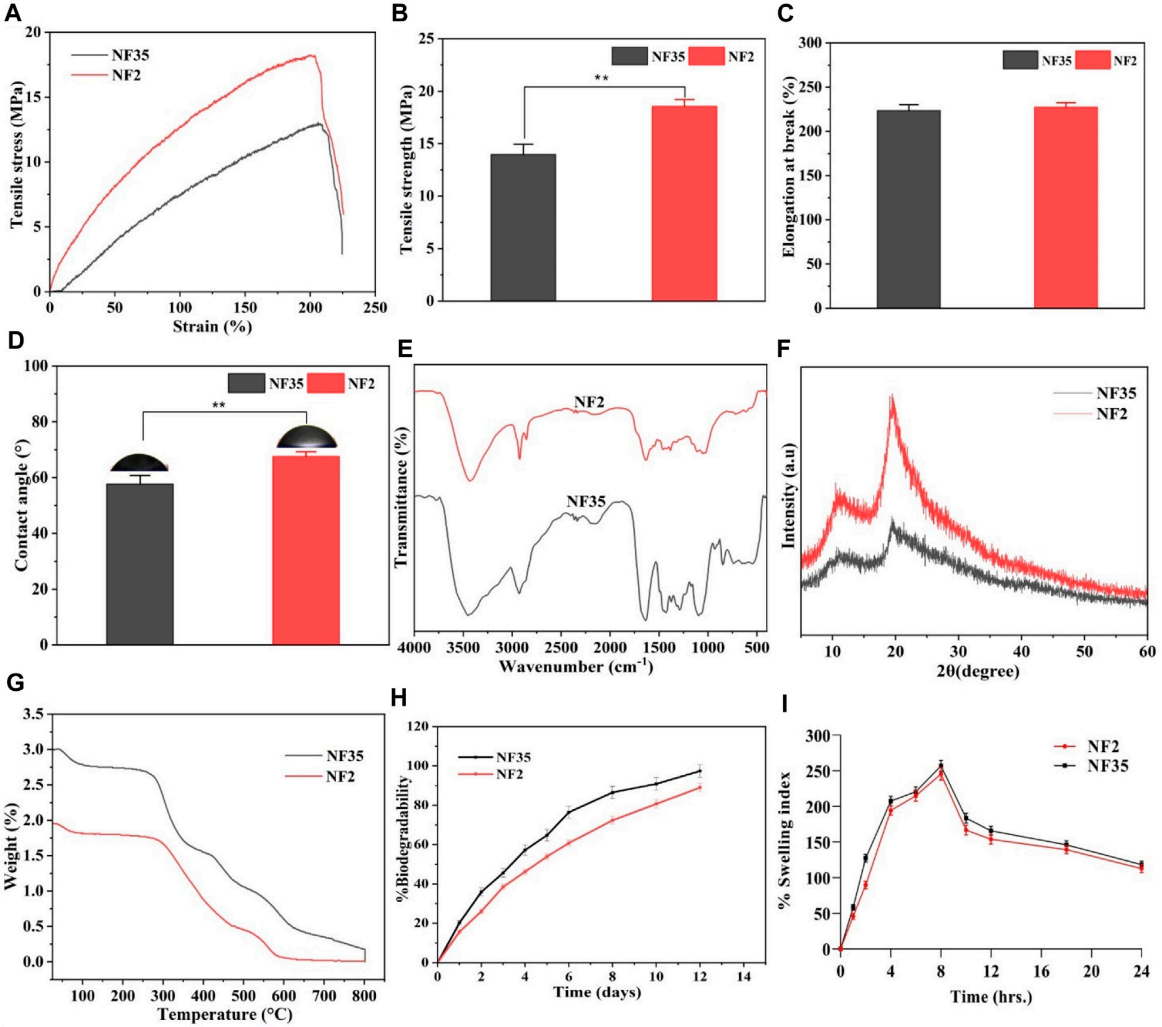
Different physiochemical properties of developed PVP/PVA nanofiber in acetic acid and ethanol solvent; **(A)** Represents the stress versus strain plot, **(B)** histogram shows the tensile strength, **(C)** shows the elongation at break (%), **(D)** contact angle measurement, **(E)** FTIR spectra, **(F)** XRD spectra, **(G)** TGA analysis plot, **(H)** *in vitro* degradability for 2 weeks and **(I)** swelling index (%) up to 24 h of (NF2 and NF35) nanofiber.

Mechanical properties hold paramount importance for nanofibers, as the underlying matrix must possess robust mechanical strength to effectively facilitate tissue repair. Evaluating the scaffolds’ tensile strength (TS) measured in MPa was accomplished through the assessment of strain-stress curves. Figures 6A–C illustrate stress-versus-strain curves and the corresponding variations in TS and elongation at break (EB) for selected scaffolds. In the case of PVP/PVA produced using an acetic acid solvent (designated as NF2), the recorded TS and EB values were 18.3 MPa and 228.06%, respectively. Similarly, for PVP/PVA synthesized with an ethanol solvent (NF35), the TS and EB values were 13.1 MPa and 224.6%, respectively (Figures 6B, C). It has been substantiated that a heightened porosity tends to adversely impact mechanical behaviour (Maheshwari et al., 2017). Considering the nanofiber diameters, a reduction in diameter correspondingly amplified the mechanical response, encompassing Young’s modulus and tensile strength. This augmentation in mechanical attributes was attributed to the constrained distribution of stress within the fibres due to the surface confinement of polymer chains. An intriguing hypothesis emerges: nanofibers characterized by uniform diameter distributions yield a consistent structure that bolsters resistance against axial tensile forces. However, the mechanical strength must strike an optimal balance, neither being excessively high nor overly low. Excessively high TS might lead to prolonged scaffold presence post-regeneration, while insufficient TS could hinder adequate cell growth and support during the critical regeneration phase.

#### 3.4.2 Contact angle

The determination of hydrophilicity and wettability of the NF2 and NF35 scaffolds involved employing the static water contact angle method through (JC 2000C1; POWEREACH China) instrument. This methodology facilitated the measurement of the contact angle for the optimized nanofiber formulations. Wettability holds pivotal importance as it significantly impacts a scaffold’s mechanical stability and its interactions with adhering cells. In the course of contact angle analysis, the shape of the liquid droplet was contingent upon variables such as liquid surface tension, gravity, and the density difference between the liquid and the nanofibers. The outcomes of the contact angle measurements, visualized in Figure 6D, showcased water droplet behaviour on the nanofiber surfaces. Notably, the abundant water content characteristic of natural polymers engenders a hydrated environment conducive to nutrient and metabolite diffusion, thus favourably influencing cellular regulatory processes.

Hydrophilicity is a key determinant of bioactivity, rendering hydrophilic scaffolds preferable for tissue engineering applications. Specifically, a surface is considered hydrophilic if the contact angle measures below 90° and hydrophobic if the angle exceeds 90°. For NF2 and NF35, the contact angles were 67.89° and 58.31°, respectively. This divergence in contact angles between NF2 and NF35 might be attributed to the influence of solvent effects and nanofiber diameter.

#### 3.4.3 FTIR analysis

Different absorption bands within 4,000–500 cm^-1^ were recorded in FTIR spectra of PVA/PVP nanofibers. Figure 6E shows FTIR spectra of the two selected nanofiber scaffolds (NF2 and NF35) obtained from acetic acid and ethanol. The FTIR spectrum of both the nanofiber showed a broad peak at 3,468 cm^-1^ along with strong intensity due to the stretching vibrations of hydroxyl group in both the nanofiber. But the intensity of peak of hydroxyl group in NF35 was more, it may be due to the presence of more hydrogen bonding in the presence of ethanol. The C-H bending at 842 cm^-1^ in PVA polymer, the band at 1,077 cm^-1^ confirm the presence of C-O vibration of PVA-PVP. In addition, the presence of a 1,631 cm^-1^ peak was due to stretching vibrations of the carbonyl group present in PVA. The band at about 1,279 cm^-1^ corresponds to C-O stretching of acetyl groups present on the PVA backbone. The appearance of C-O stretching is due to the semi-crystalline nature of the blends. A band at 1,373 cm^-1^ is attributed to C-N bond, mainly from the functional group of PVP. The vibration band at about 1,636 cm^-1^ corresponds to C-O symmetric bending of PVA and PVP. The band corresponding to CH2 asymmetric stretching vibration appeared around 2,928 cm^-1^ in PVP.

#### 3.4.4 XRD study

An XRD study was conducted to investigate electrospinning-induced crystalline changes and provide details regarding the occurrence of complex formation between different polymers. The XRD patterns of the PVP/PVA blends with different solvents are shown in Figure 6F, and data interpretation was performed using the intensities of the peaks obtained from the spectra. The sharp peaks in the pattern confirmed the crystalline nature of the polymers. The diffraction patterns of the spectra with broad halos confirmed the amorphous nature of the polymers. NF2 fabricated using acetic acid and NF35 produced with ethanol exhibited notably similar significant peaks in the PVP-PVA blend nanofibers, specifically at 2θ = 19.61° for PVP and 11.52° for PVA. Within NF2, the peak intensity experienced an augmentation compared to NF35, while the peak position remained constant. These peaks, observed for both NF2 and NF35, were characterized by short and broad profiles, indicative of the substantially amorphous nature inherent to the blend nanofibers. The observed shift in peak position in the blended samples was attributed to hydrogen bonding interactions between PVA and PVP. This phenomenon likely arises due to hydrogen bonding between the hydroxyl (OH) groups present in PVA and the carbonyl group in PVP (Yadav et al., 2022).

#### 3.4.5 TGA analysis

The thermal stabilities of the selected NF2 and NF35 nanofibers in different solvents were examined by TGA, as shown in Figure 6G. From the figure it can be seen that both the materials are thermally stable as the materials get completely decomposed at 800°C. NF35 constrains slightly better stability than the NF2 at different temperature as ∼320°C, 440°C and 590°C. This increase in temperature declares the decomposition of materials because of the degradation of polymeric side chain. Hence, both the materials were thermally stable.

#### 3.4.6 *In-vitro* degradation studies

The degree of degradation of NF 2 and NF 35 scaffold was also determined by observing the mass change of the samples after immersion in PBS. The degradation behavior of the scaffold’s during incubation is depicted in Figure 6H. Changes in electrospinning parameters cause variations in nanofiber density and diameter. Scaffolds NF2 and NF35 showed 88% and 97% degradation, after 12 days of incubation in PBS with similar patterns. Scaffolds NF2 and NF35 exhibited low and high rates of deterioration, respectively. The deterioration behavior can be influenced by several factors. The changes in the parameters of electrospinning led to differences in the density and diameter of the nanofibers.

#### 3.4.7 Swelling studies

The swelling behaviour exhibited by the scaffolds (NF2 and NF35) underscores their capacity to facilitate nutrient and waste exchange between the cellular environment and the cells embedded within the scaffold, a crucial aspect in the creation of artificial tissues. Swelling, in this context, signifies the ability to imbibe moisture and establish stability within biological systems. It offers potential as a carrier material for cell proliferation and differentiation, consequently playing a vital role in tissue engineering. Upon implantation, biomaterials interact with the surrounding fluids, initially by uptaking them, thus promoting the degradation process. The water uptake makes the materials more flexible and promotes changes in the dimensions of the implant material.

As portrayed in Figure 6I, the swelling behaviour of the selected electrospun scaffolds is depicted. Notably, hydrophilicity holds significance for tissue engineering scaffolds, as it enhances cell viability and proliferation. Despite all the scaffolds being composed of a PVP/PVA compound, variations exist in their operating parameters. Nanofiber diameter emerges as a pivotal parameter in electrospun scaffolds, influenced by factors such as surface tension, solution viscosity, working distance, flow rate, crystallization characteristics, and applied voltage. Furthermore, the nanofiber diameter’s influence extends to scaffold porosity. In light of these factors, it can be inferred that alterations in operating conditions can exert a measurable impact on the scaffold’s level of porosity (Shimko et al., 2005).

### 3.5 *In-vitro* hemocompatibility test

**FIGURE 7:**
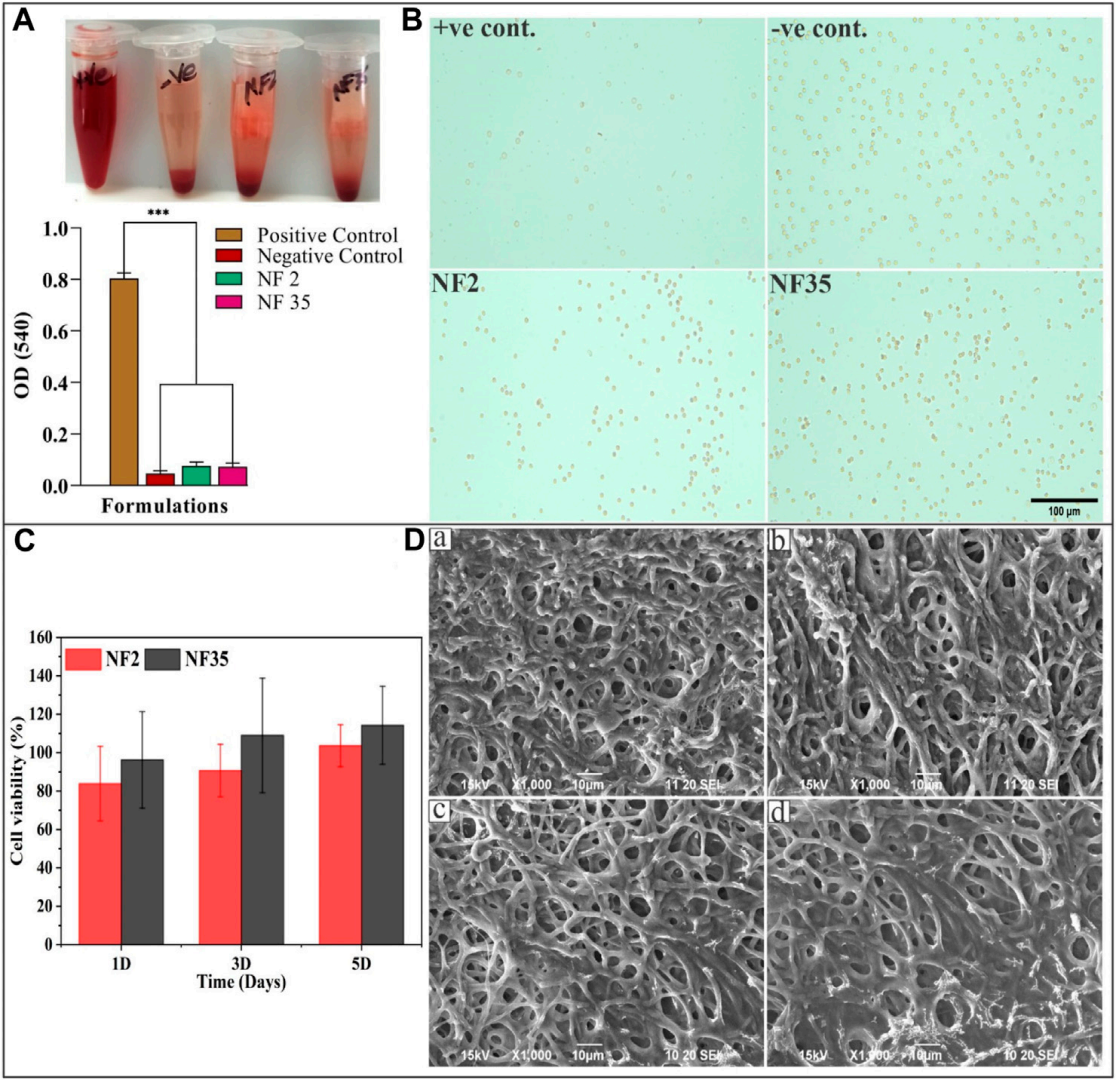
The study of hemolysis and the effect of scaffold on RBCs as well as cell viability and cytocompatibility of nanofiber; **(A)** The histogram demonstrated the absorbance value of +Ve control, -Ve control, NF2 and NF35 at 540 nm; **(B)** The microscopic image of intact RBCs status after 3 h of incubation with the control groups and nanofiber group (NF2 and NF35); **(C)** Effects of NF2 and NF35 nanofibers on cell viability of L929 cells for 1, 3 and 5 days, **(D)** SEM images of L929 cells seeded on NF2 and NF35 nanofibers after 3 days **(A,B)** and **(C,D)** presents the adhered cells after 5 days of seeding.

The hemolysis test is valuable in assessing blood compatibility as it reflects cytotoxicity. RBCs can lyse and release biomolecules like hemoglobin when they encounter water or foreign substances, due to osmotic stress. Damaged RBCs can attract platelets, accelerating coagulation and hindering tissue regeneration (Balaji et al., 2016). An optimal nanofiber dressing should preserve RBC integrity and avoid triggering coagulation while supporting tissue healing. The central focus of our study was to evaluate material compatibility with RBCs. Typically, for implanted materials, hemolysis levels below 5% are recommended. Microscopic images (Figure 7B) reaffirmed intact RBCs and nanofiber compatibility. As depicted in Figure 7A, the positive control exhibited notably higher hemolysis (few intact RBCs) compared to the negative control, NF2, and NF35. Notably, NF2 and NF35 displayed hemolysis similar to the negative control, evident by numerous intact RBCs in microscopic images. Consequently, NF2 and NF35 can be deemed highly biocompatible and non-toxic, positioning them favorably for biomedical and tissue regeneration applications.

### 3.6 *In-vitro* cell line study

#### 3.6.1 Cell viability

The impact of PVP/PVA nanofibers, specifically NF2 and NF5, on L929 fibroblast cell viability was assessed through a CCK-8 assay. The cell viability results from day 1 displayed more than 80% cell viability for NF2 and 90% for NF35 (Figure 7C). Remarkably, on days 3 and 5 of the viability assay, both nanofiber types exhibited a notable augmentation in cell viability compared to day 1. This observed trend of enhanced cell viability suggests a potential positive influence of these nanofibers on the proliferation of L929 cells over time. So, it may be concluded from above results that these nanofibers are non-toxic as well as biocompatible and significantly enhanced the cell proliferation and ascertained the potential applications of these nanofibers in promoting cell growth and viability.

#### 3.6.2 Cytocompatibility and cell adhesion test on L929 cell lines

Figure 7D show L929 cells adhesion and proliferation after treatment with prepared nanofibers on different time intervals of 3 and 5 days. The SEM photomicrographs indicate that the seeded cells are well adhered onto the surface of the scaffolds, which signifies the good biocompatibility of the scaffolds. Our results showed that nanofiber provided a good cell adhesion and proliferation property.

### 3.7 Subcutaneous implantation

**FIGURE 8:**
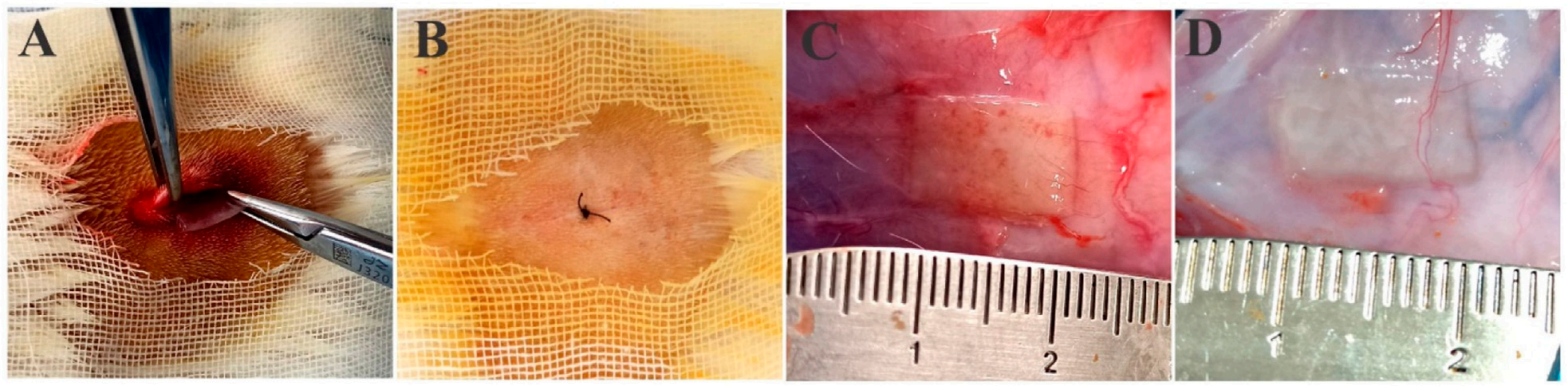
**(A)** Surgical scheme of scaffold’s subcutaneous implantation, **(B)** post-implantation suturing, **(C)** postoperative images of the scaffold NF2 and **(D)** NF35 nanofibers scaffolds remained 3 weeks after implantation.

The subcutaneous implantation of PVP/PVA nanofiber was performed in rat (Figures 8A, B), followed by careful suturing and housing the animals in a controlled environment, represented a pivotal experimental approach to investigate the *in vivo* biocompatibility and potential therapeutic applications of these nanofibers. This procedure facilitated the exploration of nanofiber-host tissue interactions, cellular responses, and overall biodegradation processes within a controlled condition. The chosen subcutaneous implantation technique, along with precise suturing and controlled animal housing, ensured a more realistic representation of the nanofibers’ interaction with the surrounding tissue. After 3 weeks of subcutaneous implantation, the nanofibers’ macroscopic appearance was similar to that of pre-implant hydrated fibre (Figures 8C, D). These studies significantly contributed to our understanding of the materials’ viability for applications in tissue engineering, wound healing, and other biomedical fields.

### 3.8 *In-vivo* biocompatibility study by (H&E) staining

**FIGURE 9:**
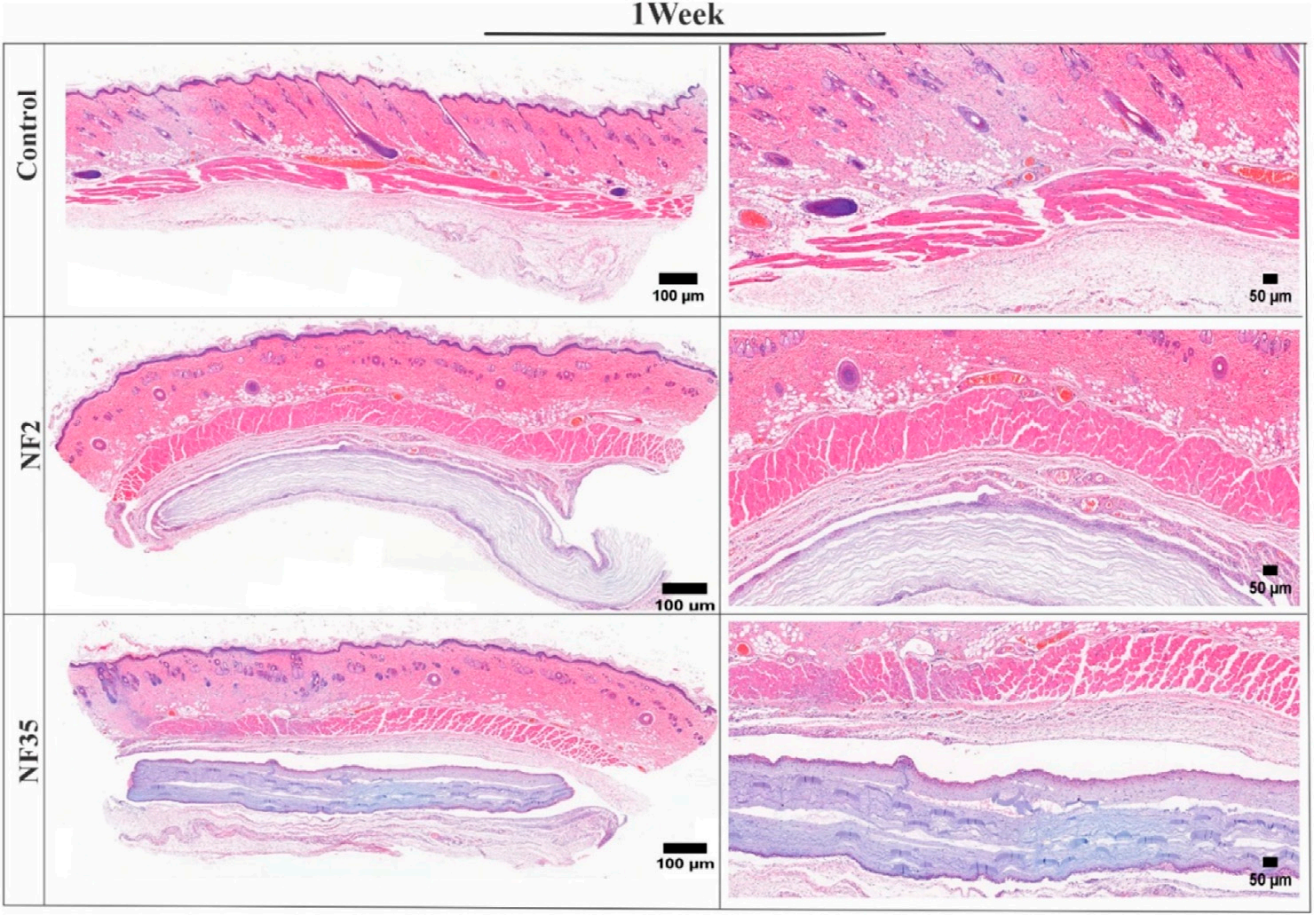
H&E-stained images of surrounding tissue of subcutaneous implanted PVP/PVA nanofiber for control, NF2 and NF35. Reconstruction of the full and interior membrane sections after 1 week of implantation.

The *in vivo* degradation of the NF2 and NF35 electrospun nanofiber was evaluated by implant tests. One week after subcutaneous implantation, the nanofibers’ macroscopic appearance was similar to that of pre-implant hydrated fibre (Figure 9). After the implantation and retrieval of the electrospun nanofiber, the specimens were routinely processed for histology, and transversal sections were analyzed by standard H&E staining.

**FIGURE 10:**
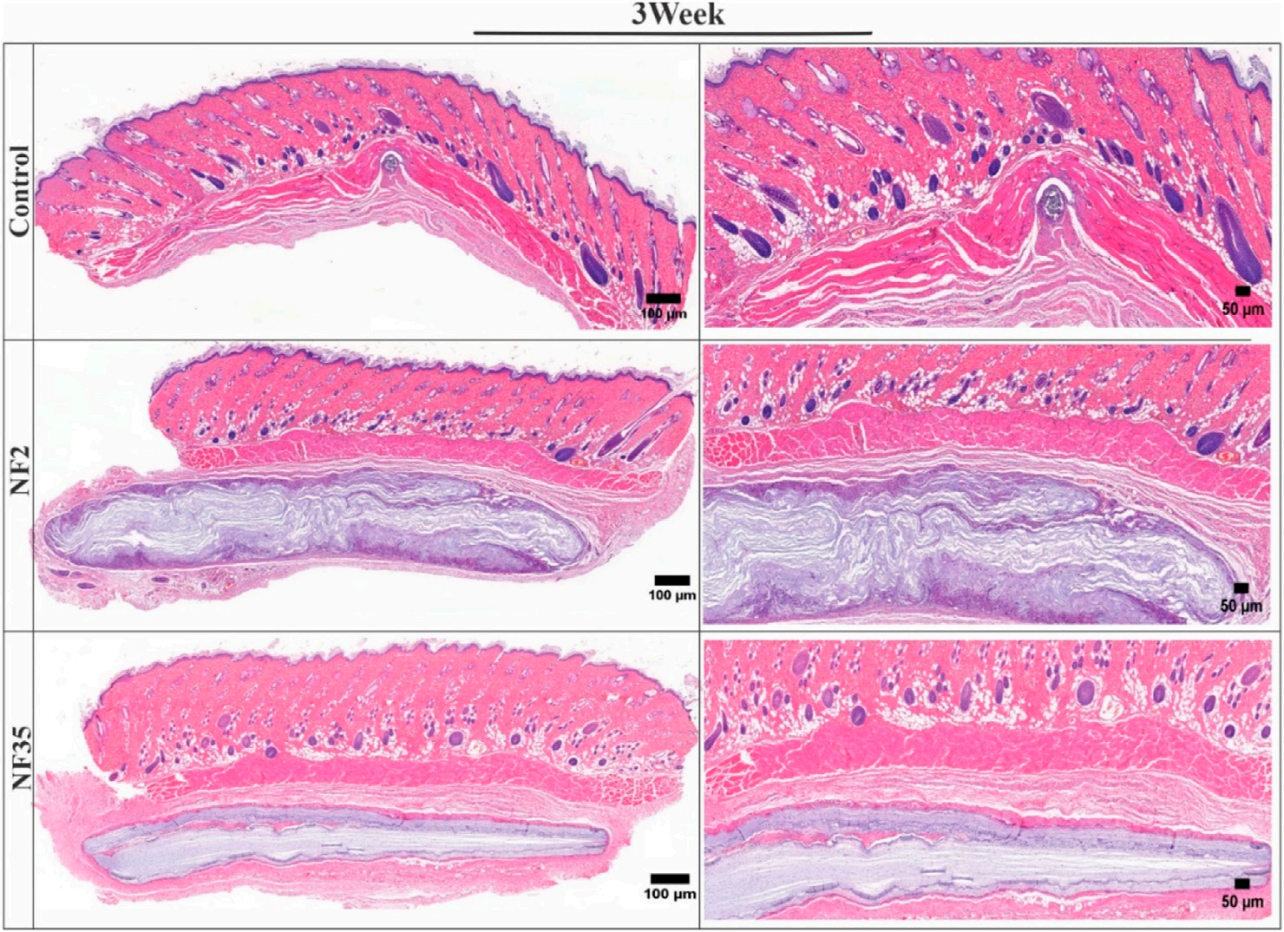
H&E-stained images of surrounding tissue of subcutaneous implanted PVP/PVA nanofiber for control, NF2 and NF35. Reconstruction of the full and interior membrane sections after 3 weeks of implantation.

The H&E-stained tissue samples collected after one and 3 weeks revealed the absence of any significant adverse reactions, inflammatory responses, or cellular infiltrations in the surrounding tissue (Figures 9, 10). This positive outcome can be attributed to the favourable interaction between the nanofibers and various cells present within the tissue. Specifically, fibroblasts play a crucial role in tissue repair and extracellular matrix formation, contributing to the nanofibers’ integration into the tissue. Additionally, the presence of macrophages and neutrophils indicates the absence of a robust immune response, further affirming the biocompatibility of NF2 and NF5. These findings suggest that the nanofibers have the potential to support tissue regeneration and therapeutic interventions, making them promising candidates for various biomedical applications.

## 4 Discussion

Accordingly, the *in vitro* and *in vivo* studies described previously regarding the non-conformable results, it is important to highlight that PVP/PVA were used with different molecular weights, different types of solvents with different concentrations, and the degradation was monitored over different periods of time. Each parameter or all together directly influence the results, compromising a correlation between the studies.

This comprehensive study successfully explored the influence of process parameters such as concentration, voltage, nozzle-to-collector distance, flow rate and solvent selections on the properties of electrospun PVP/PVA nanofibers. The CCD analysis confirmed that polymer concentration, solvent type and operating parameters were the main significant variables affecting the PVP/PVA nanofiber surface morphology. The quadratic equations derived from the DOE software were subjected to ANOVA and goodness-of-fit statistics, the summarized results of which are presented in (Supplementary Table S1). The reliability of a model is substantiated by examining the *p*-value, which, across all designs, is consistently below 0.05. This implies that the generated models are both valid and significant. Evaluating the influence of polymer concentration on nanofiber producibility, the reliability of the fitted model is assessed through the R-squared (*R* ^2^) value, along with its adjusted counterpart (adjusted *R* ^2^). In this context, a model is considered valid if its *R* ^2^ value is equal to or greater than 0.60, as affirmed by the model under consideration. The predictive capability of the model for new observations is reflected in the Predictive R-squared (Pred-R^2^), while the regular *R* ^2^ and adjusted *R* ^2^ values reflect the model’s alignment with empirical outcomes. The Pred-R^2^ and Adj-R^2^ values stand at 0.9323 and 0.9817, respectively for nanofiber producibility (Supplementary Table S1).

A preliminary series of experiments were conducted to ascertain the optimal electrospinning parameters for the PVP/PVA solution. Voltages below 8 kV led to the formation of droplets at the tip of the capillary, with no subsequent jet formation. By increasing the applied voltage to the range of 8–10 kV, the size of the hanging droplet decreased until the emergence of the Taylor cone and the stabilization of a jet between 8–10 kV. At voltages exceeding 26 kV, the jet became unstable and exhibited splitting. It was evident that a delicate equilibrium had to be maintained between the rate of solution insertion and removal at the needle’s tip.

For the electrospinning process, an appropriate flow rate within the range of 1–2.6 ml/h was determined. Higher flow rates caused an accumulation of excessive solution at the nozzle’s tip, resulting in solution dripping and the formation of wet fibers on the collector. Conversely, lower flow rates were insufficient to sustain equilibrium, leading to the disappearance of the Taylor cone and the initiation of jet formation from within the needle, ultimately interrupting the jet stream. Consequently, flow rates between 1.5–2.5 ml/h and distances of 8–12 cm were chosen as the optimal lower and upper limits, respectively. An array of 18 experiments was systematically conducted, covering the specified ranges for voltage, flow rate, and distance. The outcomes of these experiments were subsequently evaluated using RSM.

These process parameters were subsequently evaluated using ANOVA, with the summarized outcomes presented in Supplementary Tables S2, S3. The obtained *p*-values, all below 0.05, signify the reliability of all models. Another crucial indicator for assessing the models is the coefficient of determination (R-squared or *R* ^2^). This value signifies the proportion of the total variability that the regression model is able to explain. The *R* ^2^ values obtained in this study confirmed the models’ validity, as they exceeded the threshold of 0.60. Exploration of the comprehensive impact of these parameters on all produced nanofiber batches is elaborated upon in the subsequent discussion.

SEM was utilized for the morphological analysis of nanofibers, encompassing characteristics such as fiber shape, diameter, and surface structure. This inspection exposed the vital role of nanofiber topography in early cellular processes, such as adhesion and proliferation (Teixeira et al., 2004). Upon attaining optimal process parameters, the resultant non-woven fiber mats exhibited a composition of uniform, porous, and randomly aligned fibers devoid of beads.

The obtained nanofibers exhibited a consistent and controlled diameter range of 150–400 nm, indicative of a precise electrospinning process. Mechanical testing revealed notable tensile strength values ranged from 13.81 to 18.3 MPa for PVP/PVA nanofibers, highlighting their structural integrity and suitability for regenerative medicine and drug delivery applications. XRD analysis underscored the amorphous nature of the PVP/PVA nanofibers, suggesting their potential for enhanced drug loading and release kinetics. The FTIR results provided evidence of consistent and solvent-independent interactions, reinforcing the reliability and reproducibility of the fabrication process. The significant hydrophilicity exhibited by the PVP/PVA nanofibers, as evidenced by contact angle measurements, bodes well for their interactions with biological systems. This property was further corroborated by the extended water retention observed in swelling studies, aligning with the requirements of sustained and controlled drug release applications. Moreover, the 2-week biodegradation timeframe revealed through *in-vitro* degradation studies indicates that these nanofibers possess the necessary characteristics for tissue regeneration. The *in-vivo* evaluation, including subcutaneous rat implantation and histological analysis, underscored the exceptional tissue biocompatibility of the PVP/PVA nanofibers over a month-long period. Furthermore, the *in-vitro* cell lines studies indicated nanofibrous material promoted cell growth/proliferation.

## 5 Conclusion

PVP/PVA nanofiber scaffold was successfully prepared by using electrospinning method and explored the influence of process parameters such as concentration, voltage, nozzle-to-collector distance, flow rate and solvent selections on the properties of these nanofibers. Through a meticulous investigation using CCD and DOE, the optimal conditions for electrospinning of PVP/PVA nanofibers using ethanol and acetic acid were determined, leading to remarkable insights into their potential application in biomedical field. The physiochemical property of obtained nanofibers exhibited hydrophilic nature with porous structure similar to that found in native ECM. The *in-vivo* biocompatibility of the PVP/PVA nanofibers was assessed by histological analysis of surrounding tissue, which underline the exceptional tissue biocompatibility and appropriate *in-vivo* biodegradation. This outcome holds great promise for their application in tissue engineering, where biocompatibility and biodegradability are paramount.

## Data Availability

The original contributions presented in the study are included in the article/Supplementary Material, further inquiries can be directed to the corresponding authors.
