# Supplementary material for: Biomimetic electrospun nanofibrous scaffold for tissue engineering: preparation, optimization by design of experiments (DOE), in-vitro and in-vivo characterization
Source: Front Bioeng Biotechnol. 2023 Oct 31;11:1288539. doi: 10.3389/fbioe.2023.1288539 (PMC10646156; doi:10.3389/fbioe.2023.1288539)
Supplement: Supplementary file 1 [file DataSheet1.PDF]

Biomimetic electrospun nanofibrous scaffold for tissue engineering: Preparation, optimization by design of experiments (DOE), *in vitro* and *in vivo* characterization

Shabnam Anjum[1,5#], Ting Li[2#], Dilip Kumar Arya[3], Daoud Ali[4], Saud Alarifi[4], Wang Yulin[5], Zhang Hengtong[5], P.S Rajinikanth[3**], Qiang Ao[1,5*]

[1]Department of Tissue Engineering, School of Intelligent Medicine, China Medical University, Shenyang, Liaoning, 110122, China

[2]Department of Laboratory Medicine, Shengjing Hospital of China Medical University, Shenyang, Liaoning, 110004, China

[3]Department of Pharmaceutical Sciences, Babasaheb Bhimrao Ambedkar University, Vidya Vihar, Raebareli Road, Lucknow- 226025

[4]Department of Zoology, College of Science, King Saud University, Riyadh 11451, Saudi Arabia

[5]NMPA Key Laboratory for Quality Research and Control of Tissue Regenerative Biomaterial & Institute of Regulatory Science for Medical Device & National Engineering Research Center for Biomaterials, Sichuan University, Chengdu, Sichuan, 610064, China

*Corresponding Author Email: aoqiang@scu.edu.cn

**Co-corresponding Author Email: psrajinikanth222@gmail.com

#Shabnam Anjum and Ting Li contributed equally.

I. PVP/PVA concentration

Table S1. DOE Statistical parameters for PVP/PVA concentration

Source	Sum of Squares	df	Mean Square	F Value	p-value Prob > F	
Model	16.50	5	3.30	118.80	< 0.0001	Significant
A-PVP	13.50	1	13.50	486.00	< 0.0001	
B-PVA	1.50	1	1.50	54.00	0.0003	
AB	1.00	1	1.00	36.00	0.0010	
A^2	0.17	1	0.17	6.00	0.0498	
B^2	0.17	1	0.17	6.00	0.0498	
Residual	0.17	6	0.028			
Lack of Fit	0.17	3	0.056			
Pure Error	0.000	3	0.000			
Std. Dev.	0.17			**R^2**	0.9900	
Mean	2.33			**Adj R^2**	0.9817	
C.V. %	7.14			**Pred R^2**	0.9323	
				**Adeq Precision**	36.062	

The model quadratic equations generated for nanofiber producibility is as below.

$$\boldsymbol{Nanofiber\ Producibility} = +2.08 + 1.50 * A + 0.5000 * B + 0.5000 * AB + 0.2500 * A^2 + 0.2500 * B^2$$

Table S2. One-way ANOVA analysis for response surface quadratic model for average nanofiber diameter in acetic acid solvent

Source	Sum of Squares	df	Mean Square	F-Value	P value Prob > F	
Model	42644.10	9	4738.23	92.74	< 0.0001	Significant
A-Flow rate	15158.56	1	15158.56	296.69	< 0.0001	
B-Voltage	1434.63	1	1434.63	28.08	0.0007	
C-Distance	83.69	1	83.69	1.64	0.2365	
AB	7221.98	1	7221.98	141.35	< 0.0001	
AC	65.41	1	65.41	1.28	0.2906	
BC	2077.22	1	2077.22	40.66	0.0002	
A^2	730.29	1	730.29	14.29	0.0054	
B^2	7410.86	1	7410.86	145.05	< 0.0001	
C^2	14301.40	1	14301.40	279.91	< 0.0001	
Residual	408.74	8	51.09			

Lack of fit	408.74	5	81.75
Pure Error	0.000	3	0.000

Std. Dev.	7.15	**R^2**	0.9905
Mean	194.74	**Adj R^2**	0.9798
C.V. %	3.67	**Pred R^2**	0.9496
		**Adeq Precision**	31.0164

The model quadratic equations generated for nanofiber diameter in acetic acid solvent is as below.

$$\boldsymbol{Diameter}\ (\boldsymbol{nm}) = +196.92 - 38.93 * A + 11.98 * B + 2.89 * C + 30.05 * AB - 2.86 * AC + 16.11 * BC + 16.42 * A^2 + 52.30 * B^2 - 72.65 * C^2$$

Table S3. One-way ANOVA analysis for response surface quadratic model for average nanofiber diameter in ethanol solvent

Source	Sum of Squares	df	Mean Square	F-Value	p-value Prob > F	
Model	1.350E+005	9	14999.82	23.07	< 0.0001	significant
A-Flow rate	13187.14	1	13187.14	20.28	0.0020	
B-Voltage	6486.50	1	6486.50	9.98	0.0134	
C-Distance	5493.49	1	5493.49	8.45	0.0197	
AB	27819.33	1	27819.33	42.79	0.0002	
AC	1407.71	1	1407.71	2.17	0.1794	
BC	3458.33	1	3458.33	5.32	0.0500	
A^2	71172.29	1	71172.29	109.48	< 0.0001	
B^2	538.69	1	538.69	0.83	0.3893	
C^2	15620.11	1	15620.11	24.03	0.0012	
Residual	5200.97	8	650.12			
Lack of Fit	5200.96	5	1040.19			
Pure Error	2.269E-003	3	7.562E-004			

Std. Dev.	25.50	**R^2**	0.9629
Mean	236.63	**Adj R^2**	0.9212

C.V. %	10.78	**Pred R^2**	0.7002
		**Adeq Precision**	15.718

The model quadratic equations generated for nanofiber diameter in ethanol solvent is as below.

$$\boldsymbol{Diameter}\ (\boldsymbol{nm}) = +196.61 + 36.31 * A - 25.47 * B + 23.44 * C + 58.97 * AB + 13.27 * AC + 20.79 * BC + 162.07 * A^2 - 14.10 * B^2 - 75.92 * C^2$$
